# Radiation-Induced Synthesis of Asymmetric Porous PVDF-g-PIL Membranes via β-Cyclodextrin Leaching for Vanadium Redox Flow Battery

**DOI:** 10.3390/ma19030583

**Published:** 2026-02-03

**Authors:** Jiangtao Yu, Wenkang Li, Wei Niu, Manman Zhang, Junqing Bai, Pengtao Li, Liang Wang, Yuqing Cui, Shuanfang Cui, Xueyan Que, Jun Ma, Long Zhao

**Affiliations:** 1 School of Nuclear Science and Technology, University of Science and Technology of China, Hefei 230026, China; yujiangtao@ssnhsit.com; 2 Yangling Hesheng Irradiation Technologies Co., Ltd., Yangling, Xianyang 712000, China; niuwei@ssnhsit.com (W.N.); baijunqing@ssnhsit.com (J.B.); lipengtao@ssnhsit.com (P.L.); wangliang@ssnhsit.com (L.W.); cuiyuqing@ssnhsit.com (Y.C.); cuishuanfang@ssnhsit.com (S.C.); 3 State Key Laboratory of Advanced Electromagnetic Technology, School of Electrical and Electronic Engineering, Huazhong University of Science and Technology, Wuhan 430074, China; liwenkang@hust.edu.cn (W.L.); zhangmm@hust.edu.cn (M.Z.)

**Keywords:** PVDF porous membrane, irradiation grafting, vanadium flow battery, β-cyclodextrin

## Abstract

This study aims to address the limitations of dense polyvinylidene fluoride (PVDF) membranes grafted with vinyl ethyl imidazole tetrafluoroborate, which exhibit low hydrophilicity and ionic conductivity in vanadium redox flow batteries (VRFBs). To improve these properties, water-soluble β-cyclodextrin was introduced as a porogen to fabricate asymmetric porous membranes. The porous structure was controlled by varying the porogen content (10–50 wt%), and the resulting membranes were characterized using FTIR, SEM, TGA, and electrochemical tests. This unique architecture led to a significant enhancement in ionic conductivity (to 71.69 mS/cm, from 6.73 mS/cm for the dense membranes), porosity (up to 40.24%), and water uptake (up to 31.8%), while maintaining robust mechanical strength (tensile strength 14.96 MPa) suitable for VRFB assembly and operation. In single-cell performance tests across a range of current densities, clear trends emerged: Coulombic efficiency (CE) decreased with higher porosity, whereas voltage efficiency (VE) followed the opposite trend. Consequently, the optimal energy efficiency (EE) was achieved with the intermediate porogen content, successfully balancing conductivity and selectivity. This work demonstrates a green and scalable approach to developing high-performance porous membranes for VRFB applications.

## 1. Introduction

Due to the intermittent nature of renewable electricity, this form of power cannot be used directly [1,2,3]. A practical solution involves its stabilization through energy storage systems [4]. VRFBs represent a particularly promising technology for grid-scale power regulation among available storage options. They offer several notable advantages, including large capacity, rapid charge–discharge capability, design flexibility, high safety, long operational lifespan, and satisfactory efficiency [5,6,7].

A key component of VRFBs is the ion exchange membrane (IEM), which separates the positive and negative electrolytes while allowing charge-carrying ions to pass. An ideal IEM should combine high ionic conductivity, low vanadium ion crossover, good chemical stability, and robust mechanical properties [8,9,10]. Currently, Nafion stands as the most commercially utilized ion exchange membrane (IEM) in vanadium redox flow batteries (VRFBs), valued for its strong ionic conductivity and mechanical strength [11,12]. However, its widespread application and development in VRFBs are significantly hindered by two major disadvantages: prohibitively high cost and substantial vanadium ion crossover [7,13,14]. An alternative approach involves the use of anion exchange membranes (AEMs) functionalized with specific cationic groups [15,16]. These membranes demonstrate exceptionally low vanadium permeability, a characteristic attributed to the powerful “Donnan” exclusion effect [17,18,19]. A common limitation of AEMs, nevertheless, is their often unsatisfactory ion conductivity, primarily resulting from the large Stokes radius of the transported anions, such as SO_4_^2−^ and HSO_4_^−^ [20]. Within the category of AEMs, those based on imidazolium groups exhibit a certain degree of intrinsic proton conductivity [17,21,22]. This property arises from the presence of a lone electron pair on the imidazolium functional group, which can act as a proton acceptor. A well-established strategy for improving the ion conductivity of these imidazolium-based membranes is to increase their acid doping level [23]. For instance, Che and colleagues fabricated a series of porous polybenzimidazole (PBI) membranes using an SiO_2_ templating method [24]. This introduced porous architecture substantially elevated the membranes’ capacity for acid absorption, consequently enabling the modified PBI membranes to achieve a significantly lower area resistance than that of Nafion115. Furthermore, the incorporation of specific additives can also be an effective means of enhancing the acid-doping level. In a study by Devrim et al. [25], the integration of sulfonated graphene oxide into a PBI matrix led to the creation of composite membranes that exhibited both a higher acid uptake and superior proton conductivity, thereby improving key performance metrics.

In the context of VRFBs, porous IEMs have demonstrated advantages in balancing conductivity and selectivity. A study by Victor E. Sizov and colleagues [26] described the fabrication of porous PBI membranes through non-solvent-induced phase separation within supercritical CO_2_, achieving higher energy efficiency than Nafion. Similarly, Luo and collaborators [27] produced PBI porous membranes via water vapor-induced phase separation, achieving notable selectivity between proton and vanadium ion transport. Wang’s group [28] proposed the fabrication of PTFE/silica porous membranes by integrating silica particles, thereby merging outstanding chemical stability with cost-effectiveness. The use of pore-forming agents represents another viable route for creating porous membranes. For example, Matsuyama et al. [29] investigated the effect of polyvinylpyrrolidone (PVP) on the porous morphology of polysulfone membranes. In a related study, Li’s team [30] effectively synthesized porous polyethersulfone membranes by adding PVP and applying phase transition methods. However, many existing porous membranes exhibit symmetric or randomly distributed pores, which may not optimize the trade-off between low resistance and high selectivity.

Building on these advances, we propose an asymmetric porous AEM, larger pores on one side and smaller pores on the other, to further decouple and enhance these competing properties. The larger-pore side can reduce ion transport resistance and improve electrolyte wetting, while the denser small-pore side can strengthen Donnan exclusion and limit vanadium crossover. Such architecture has the potential to simultaneously achieve high conductivity and excellent selectivity, a combination difficult to attain with conventional symmetric membranes. The incorporation of β-cyclodextrin into imidazole-functionalized polymers, coupled with a controlled phase-inversion process, enables the fabrication of AEMs exhibiting an asymmetric pore morphology, yielding a membrane with a pore-size gradient: a more open morphology on the air-exposed side and a tighter structure on the glass side. This method is simple, scalable, and environmentally benign. β-Cyclodextrin is an environmentally benign and biodegradable compound, synthesized through the enzymatic processing of starch—a renewable resource. It presents no threat to ecological systems or human health. The compound can be readily washed away with aqueous solutions, circumventing the need for organic solvents, acidic treatments, or highly corrosive reagents, and thereby avoiding the production of dangerous liquid effluents. Both its synthesis and its environmental breakdown are consistent with the tenets of green chemistry.

Based on previous work by the group, a dense anion exchange membrane composed of PVDF-IL was developed, demonstrating outstanding performance in blocking vanadium ions [31]. However, its ionic conductivity remained limited by the characteristics of the sulfate groups responsible for ion transport. To address this limitation, the current work focuses on creating a porous structure within the dense membrane matrix. This structural modification reduces the thickness of the separation layer and increases the number of acid–base interactions between HSO_4_^−^ and imidazolium cations, thereby facilitating ion transport and enhancing overall ionic conductivity. A water-soluble porogen, β-cyclodextrin, which does not dissolve in organic solvents, was mixed with PVDF-IL powder in a DMF solution to produce the porous membrane. Following the film-casting procedure, the template was efficiently extracted by immersing the membrane in deionized water. The successful implementation of this environmentally friendly and scalable pore-forming technique was confirmed through various characterization methods, including FTIR, SEM and TGA. Additionally, the functional performance of the membranes was evaluated based on key metrics such as mechanical properties, water uptake rate, ion conductivity, vanadium permeation resistance and overall performance within a vanadium redox flow battery system.

## 2. Experimental

### 2.1. Materials

Polyvinylidene fluoride powder (Solef 6020) was sourced from SOLVAY Inc. (Houston, TX, USA). The compound 1-vinyl-3-ethyl imidazolium tetrafluoroborate ([C_2_VIm][BF_4_]) was supplied by the Lanzhou Institute of Chemical Physics (Lanzhou, China). Dimethylformamide (DMF), β-cyclodextrin and other general chemical reagents were procured from Sinopharm Chemical Reagent Co., Ltd. (Shanghai, China), while vanadium sulfate (VOSO_4_) was provided by Wuxi Zhanwang Chemical Reagent Co., Ltd. (Wuxi, China). A Nafion 115 membrane was supplied by DuPont (Wilmington, DE, USA). Every chemical was employed in its received state, with no additional purification steps undertaken.

### 2.2. Preparation of PVDF-IL Powder

The materials were primarily synthesized using a pre-irradiation technique (Wasik Associates, Dracut, MA, USA). A measured amount of PVDF powder was first enclosed in a polytetrafluoroethylene bag, which was then evacuated under vacuum to avoid any interaction between oxygen and the subsequently generated active free radicals. An electron accelerator was utilized to deliver a predetermined radiation dose to the base material, thereby activating the PVDF to produce these radicals. To maintain their reactivity, the irradiated PVDF powder was immediately stored in dry ice. This activated powder was subsequently introduced into an aqueous solution of vinyl ethylimidazolium tetrafluoroborate ionic liquid, prepared beforehand at a set concentration. The mixture was maintained at 60 °C for six hours to accelerate the reaction while simultaneously minimizing radical oxidation. A key advantage of this procedure is that the ionic liquid itself is not exposed to irradiation. Since the vinyl ethylimidazolium tetrafluoroborate monomer features unsaturated bonds, the production of self-polymerized by-products is substantially limited. This effectively minimizes impurities and ensures the successful progression of the chemical grafting reaction. After grafting, the resulting powder underwent thorough cleaning via suction filtration with deionized water and ethanol, continuing until the filtrate appeared clear. The final PVDF-IL powder achieved in this experiment had a grafting rate of 30%.

### 2.3. Preparation of PVDF-IL Porous Membranes

A cleaned 50 mL beaker was placed on an electronic balance and tared after display stabilization. Precisely 0.5 g of PVDF-IL powder (30% grafting ratio) was measured into the vessel. Successive additions of β-cyclodextrin were made in quantities corresponding to 10–50% of the polymer mass (0.05 g to 0.25 g). Dimethylformamide was added dropwise until the total mass reached 10 g, yielding 5 wt% polymer solutions containing varying porogen concentrations (10–50%). A sterilized stirring bar was introduced, and the container was immediately sealed with plastic film to prevent atmospheric moisture absorption—a critical precaution since β-cyclodextrin exhibits high hydrophilicity and water ingress would compromise pore formation. The sealed system underwent continuous mixing at 400 rpm and 60 °C for 24 h to ensure homogeneous dissolution and porogen distribution. The resulting solution was cast onto a glass substrate with square-shaped groove and transferred to a leveled 60 °C oven for solvent evaporation. Special attention was required during this phase as β-cyclodextrin modification alters the solution’s mechanical characteristics, potentially causing film deformation during extended thermal treatment. Membrane integrity was preserved through continuous monitoring and immediate removal upon complete drying. The cured films were delicately separated from the substrates and immersed in 100 mL deionized water for 48 h, enabling porogen dissolution and consequent pore formation. Finally, the porous membranes were sectioned and treated in 3 mol/L sulfuric acid (100 mL, 72 h) to facilitate protonation.

### 2.4. Characterization

Fourier-transform infrared (FT-IR) spectroscopy was carried out on a Bruker VERTEX 70 spectrometer (Bruker Optik GmbH, Ettlingen, Germany), scanning a spectral region from 400 to 4000 cm^−1^ with a resolution set to 2 cm^−1^. To examine membrane surface morphology, field emission scanning electron microscopy (SEM) was employed using a Hitachi SU8010 system (Hitachi High-Tech Corporation, Tokyo, Japan). The thermal properties of the synthesized powder were evaluated by thermogravimetric analysis (TGA) on a Discovery TGA55 instrument (TA Instruments, New Castle, DE, USA). For this analysis, samples were heated from ambient temperature up to 800 °C at a constant rate of 10 °C per minute under a nitrogen atmosphere. The mechanical properties of both the pristine PVDF membrane and the fabricated AEMs were determined with a SEMtester 100 (Milliren Technologies Inc., Newburyport, MA, USA), applying a 100 N load. All membranes were cut into standardized strips measuring 5 cm by 1 cm prior to testing.

### 2.5. Ion Exchange Capacity, Porosity, Water Uptake, Swelling Ratio and Ion Conductivity of the PVDF-IL-CD

The ion exchange capacity (IEC) was determined through an acid-base titration technique. First, dried membrane samples were allowed to equilibrate in a 0.5 M sodium hydroxide (NaOH) solution for a period of 24 h. Subsequently, the resulting solution was titrated in reverse using a 0.5 M hydrochloric acid (HCl) solution to reach a neutral pH endpoint. The IEC value was then calculated based on the titration data, utilizing Equation (1).

(1) $$\mathrm{IEC}=\frac{C(\mathrm{NaOH})\;\times \;V(\mathrm{NaOH})\;-\;C(\mathrm{HCl})\;\times \;V(\mathrm{HCl})\;}{W_{\mathrm{dry}}}$$

where C(HCl) and C(NaOH) are the concentrations (mol L^−1^) of the initial HCl and NaOH solutions, respectively; V(HCl) and V(NaOH) are the volumes of the used HCl solution by back titration and the initial NaOH solution, respectively; and W_dry_ is the weight (g) of the dry membrane.

Pore volume is defined as the ratio of the total volume of pores within a porous membrane to the external volume of that membrane. It serves as a performance indicator for evaluating the structural integrity of a membrane. A higher pore volume indicates a greater diameter or number of pores within the membrane, resulting in lower structural integrity. In membrane performance evaluation, porosity influences multiple properties. Firstly, higher porosity facilitates ion conduction. Simultaneously, increased pore diameter or quantity diminishes vanadium resistance. Secondly, porosity affects mechanical properties: greater porosity leads to looser membrane structure, weaker mechanical strength, and increased susceptibility to rupture. To determine the membrane porosity, a 1 cm × 1 cm sample is first vacuum-dried at 60 °C for 24 h. After complete drying, the dry mass (*M_dry_*), length (*L_dry_*), width (*C_dry_*), and thickness (*D_dry_*) are immediately recorded, following the same precautions used in the water uptake test. The sample is then immersed in deionized water for 24 h. Once fully hydrated, remove it and carefully blot surface moisture with absorbent paper (following the same precautions as for wet membrane mass measurement in the water absorption test). Immediately measure its wet membrane mass (*M_wet_*).

The porosity calculation formula is shown in Equation (2):

(2) $$\varphi =\frac{M_{wet}-M_{dry}}{\rho_{w}L_{dry}C_{dry}D_{dry}}\times 100\%$$

where *φ* is the membrane porosity; *ρ_w_* is density of water at room temperature (0.997 g/cm^3^). Equation (2) calculates an apparent porosity based on the dry membrane volume (*L_dry_*, *C_dry_*, and *D_dry_*, correspond to the length (cm), width (cm), and height (cm) of the membrane, respectively). It assumes the absorbed water (mass *M_wet_* − *M_dry_*) with a density of 0.997 g/cm^3^ occupies the pore volume within the dry membrane dimensions. This provides a conservative, comparative measure of the pore space created by porogen leaching.

To determine the water uptake (WU), a gravimetric analysis was performed. The dry mass of each membrane was first recorded. Subsequently, the samples were submerged in deionized water and allowed to soak at ambient temperature for 24 h. Following this hydration period, the membranes were removed from the aqueous medium. Any residual surface moisture was carefully blotted away, and the hydrous membranes were immediately reweighed to obtain their wet mass. The WU ratio was then calculated from these mass values using Equation (3).

(3) $$\mathrm{WU}=\frac{W_{\mathrm{wet}}\;-\;W_{\mathrm{dry}}}{W_{\mathrm{dry}}}\times \;100\%$$

where W_dry_ and W_wet_ are the weights (g) of the membranes before and after soaking.

The swelling ratio (SR) was ascertained through a volumetric methodology. The initial dimensions—specifically the length, width, and thickness—of every membrane sample were measured. These specimens were then fully submerged in deionized water and left to soak for a period of 24 h under ambient conditions. Post-hydration, the membranes were extracted from the water. After meticulously blotting any superficial liquid, their swollen dimensions were promptly measured again. The SR was subsequently computed from the observed change in volume by applying Equation (4).

(4) $$\mathrm{SR}=\frac{l_{2}\;\times {\;d}_{2}\;\times \;h_{2}-{\;l}_{1}\;\times \;d_{1}\;\times {\;h}_{1}}{l_{1}\;\times {\;d}_{1}\;\times {\;h}_{1}}\;\times \;100\%$$

where l_1_, d_1_, and h_1_ represent the length, width, and height of the membrane before immersion treatment, and l_2_, d_2_, and h_2_ represent the corresponding dimensions after immersion treatment.

To characterize the ion conductivity of the PVDF-IL-CD IEM, electrochemical impedance spectroscopy (EIS) was conducted utilizing a CHI604 electrochemical workstation. An alternating current (AC) perturbation of 5 mV was applied across a frequency spectrum ranging from 0.1 to 10^5^ Hz. Before the EIS recording, the membrane underwent a 24 h acidification treatment and was subsequently secured between a pair of Cu electrodes (Figure S1). The ion conductivity (σ) was then derived from the impedance data based on the calculation provided in Equation (5).

(5) $$\sigma =\frac{L}{\mathrm{RS}}$$

where L is the thickness (cm) of the membrane, S is the contact area (cm^2^) between the membrane and the copper sheets, and R is the impendence (Ω) of the IEMs.

### 2.6. Vanadium Permeability

The vanadium ion permeability (P) of the prepared IEMs and a Nafion 115 reference was assessed using a diffusion cell fabricated from PTFE. This apparatus consisted of two 50 mL half-cells, each featuring a 1.5 cm diameter circular opening. Prior to the evaluation, the IEMs were protonated by soaking them in a 3 M H_2_SO_4_ solution for 6 h. A membrane was then securely clamped between the two halves of the cell. One reservoir was charged with 50 mL of a solution containing 1.5 M VOSO_4_ in 3 M H_2_SO_4_, and the opposing reservoir was filled with 50 mL of 1.5 M MgSO_4_ dissolved in an identical 3 M H_2_SO_4_ medium. At predetermined time intervals, aliquots were extracted from the reservoir containing MgSO_4_ to monitor the temporal change in vanadium concentration. The quantity of vanadium ions present in these samples was quantified using an Agilent 5110 ICP-OES instrument (Agilent Technologies, Inc., Santa Clara, CA, USA) (inductively coupled plasma optical emission spectrometer). The permeability coefficient was ultimately determined by applying the data to Equation (6).

(6) $$P=\frac{\mathrm{LV}}{A(C_{a}\left(t\right)\;-\;C_{b}\left(t)\right)}(\frac{d(C_{b}(t))}{dt})$$

where L is the thickness (cm) of the membrane, V is the volume (mL) of the left cell, A is the effective area (cm^2^) of the membrane, and *C*_a_(*t*) and *C_b_*(*t*) are the concentrations (mol L^−1^) in the left cell and right cell at time *t* (min), respectively. P is the vanadium permeability of the membrane.

### 2.7. Antioxidant Testing

The mass change method for evaluating antioxidant performance should be conducted according to the following steps: Cut a pretreated membrane fragment (5 cm × 5 cm) and place it in an oven (105 °C to 110 °C) to dry until the difference in mass between two consecutive weightings is less than 0.2 mg. Weigh the dried fragment using a balance to obtain its initial mass *m*_0_. Place the dried membrane fragment into a beaker, add prepared Fenton’s reagent to ensure complete immersion of the membrane, and then heat the beaker in a 60 °C water bath and maintain the temperature for 3 h. Remove the membrane and place it in a beaker containing 3 M dilute sulfuric acid solution. Soak for 30 min while gently agitating the beaker. Rinse the membrane sample with deionized water until the pH of the rinse water reaches neutrality. Place the thoroughly rinsed membrane into an oven (105–110 °C) for re-drying until the mass difference between two consecutive weightings is less than 0.2 mg. Weigh the mass using a balance to obtain *m*_1_. Compare the mass change of the dried membrane before and after Fenton reagent treatment using Formula (7) to characterize the membrane’s antioxidant performance. A higher value indicates more severe degradation of the polymer membrane during Fenton reagent treatment and poorer antioxidant properties. The antioxidant coefficient is denoted as *S_fm_*.

(7) $$S_{fm}=\frac{m_{0}-m_{1}}{m_{0}}\;\times \;100\%$$

## 3. Results and Discussion

### 3.1. Grafting Pattern of IL on PVDF

**Figure 1:**
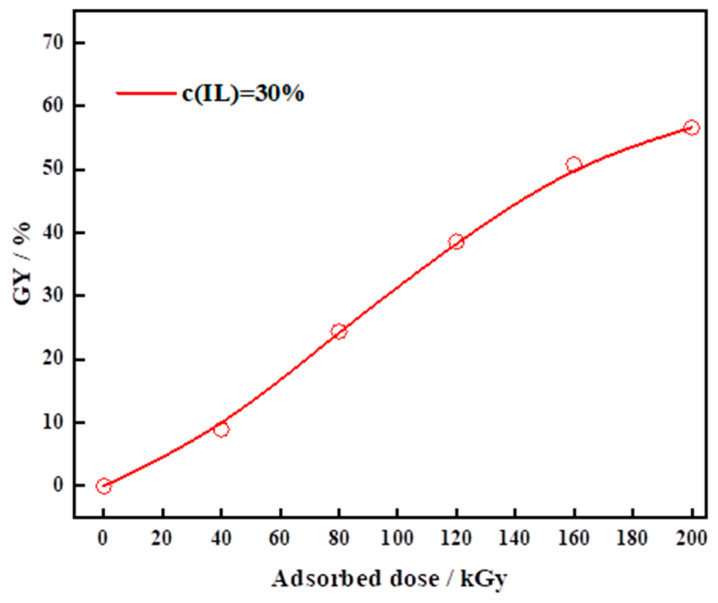
Relationship curve between grafting rate and irradiation absorbed dose at a 30% monomer concentration.

The synthesis of PVDF-IL powder was conducted following the aforementioned procedure. Building on foundational work from the group, an ionic liquid monomer concentration of 30 wt% by mass was chosen for this investigation to balance performance with cost-effectiveness. The influence of varying levels of irradiation absorption dose on the degree of grafting was also examined. The experimental data, illustrated in Figure 1, indicate a steady rise in the grafting rate as the absorbed radiation dose escalates. Beyond a threshold of 160 kGy, however, the rate of this increase diminishes. A plausible explanation is that elevated irradiation levels produce a greater number of reactive sites on the PVDF backbone, which can interact more completely with the available ionic liquid monomer. Beyond a radiation dose of 160 kGy, monomer availability for reaction with generated free radicals becomes limited, or self-polymerization occurs among the monomers. As a result, the grafting efficiency stabilizes and ceases to increase. These irradiation trials demonstrate that by modulating the absorbed dose at a fixed 30% monomer concentration, modified substrates with grafting ratios from 0% to 60% can be reliably produced, finalizing a dependable protocol for PVDF-IL synthesis.

### 3.2. Characterization of the Prepared PVDF-IL-CD IEM

**Figure 2:**
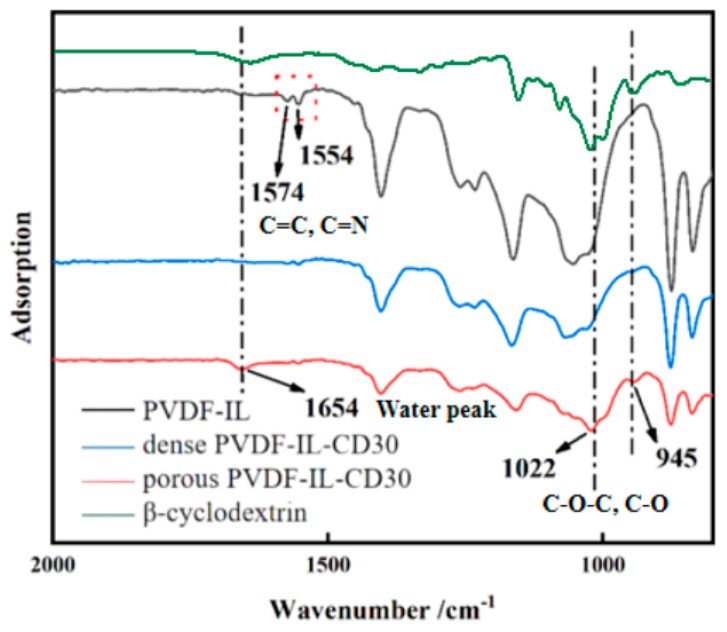
FTIR spectra of PVDF-IL-CD30 before and after removal of the pore-forming agent compared to the base material and β-cyclodextrin.

FTIR spectroscopy was employed to examine the structural variations between pristine PVDF-IL membranes and their porogen-added counterparts following aqueous immersion, with a specific focus on samples containing 30% porogen. The resulting spectra are presented in Figure 2. The analysis identified distinctive spectral features at 1554 cm^−1^ and 1574 cm^−1^, which are attributable to the imidazole ring, thereby confirming that the grafting reaction proceeded successfully. Upon the addition of cyclodextrin as a pore-forming agent, a new absorption band emerges at 1654 cm^−1^, signifying the presence of water molecules trapped within the cyclodextrin cavities. Additionally, peaks observed at 1022 cm^−1^ and 945 cm^−1^ correspond to the C-O-C stretching vibrations of the cyclodextrin structure [32]. Subsequent to the removal of the template by immersion in deionized water, the infrared spectrum of the porous membrane shows that these characteristic peaks (945 cm^−1^, 1022 cm^−1^, and 1654 cm^−1^) are almost entirely absent [33]. This observation provides confirmation that the pore-forming agent was effectively extracted, verifying the successful fabrication of the porous membrane.

**Figure 3:**
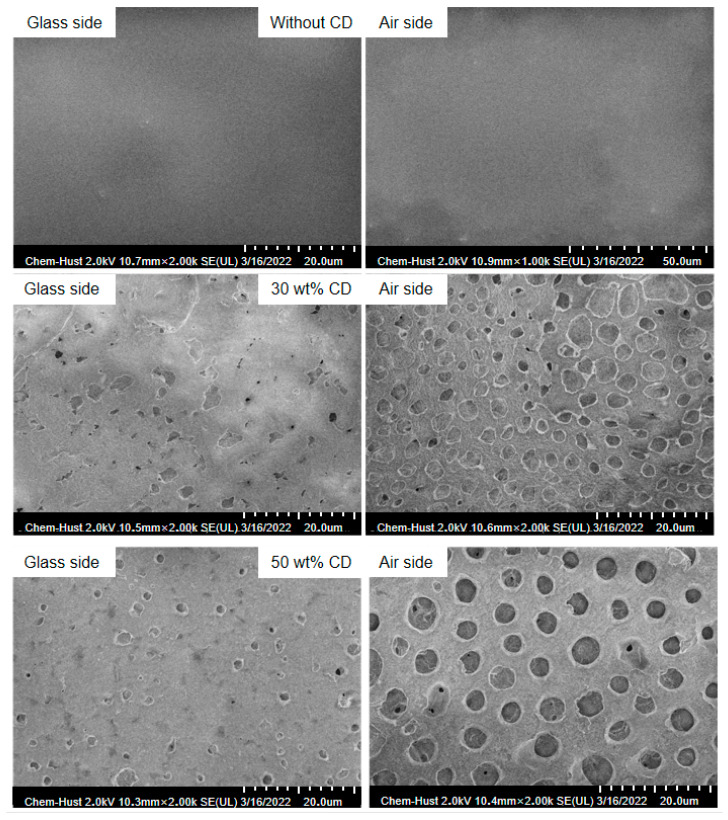
Surface structure of PVDF-IL membranes prepared with different pore-forming agents.

To investigate the morphological features of the developed asymmetric porous membranes, scanning electron microscopy (SEM) was utilized to characterize samples containing 0%, 30%, and 50% porogen. The resulting micrographs, displayed in Figure 3, show that the membrane without any pore-forming agent displayed a non-porous, compact structure on its air-facing and glass-facing surfaces. A comparative assessment of the surfaces for the 30% and 50% porogen membranes reveals that on the air side, the pore dimensions progressively expanded with greater porogen loading, growing from 1–2 μm to 4–5 μm. Concurrently, the pore population diminished, indicating that a higher concentration of porogen yields a smaller specific surface area, an effect potentially caused by aggregation of the porogen material. In contrast, the glass side of both modified membranes showed a sparse distribution of voids, a characteristic that is advantageous for preserving the material’s vanadium-blocking performance. A cross-sectional view of the membrane is shown in Figure S1. This asymmetric structure is the physical foundation for the proposed functional mechanism. The dense skin layer provides the primary barrier for vanadium ion selectivity (Donnan exclusion enhanced by smaller pores), while the macro-porous support layer facilitates rapid electrolyte wetting and reduces overall ionic resistance. The distinct porosity contrast between the two surfaces further confirms the successful fabrication of an asymmetric porous PVDF-IL membrane. The specific surface area (S_BET_) was determined via nitrogen adsorption–desorption experiments. Experimental results showed that the specific surface areas of PVDF-IL-CD30 and PVDF-IL-CD50 were 0.590 m^2^/g and 1.541 m^2^/g, respectively (Figure S2). This increase directly correlates with the development of a more extensive internal porous network as the porogen content rises, providing more interfacial area for electrolyte interaction. The increased specific surface area correspondingly facilitates greater water uptake and enhanced ionic conductivity.

**Figure 4:**
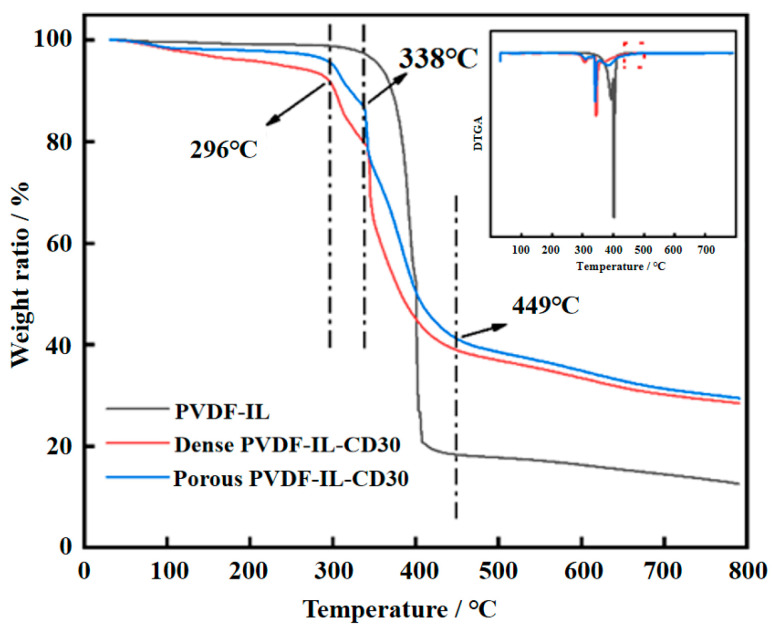
TGA test curves of PVDF-IL-CD30 before and after removal of the pore-forming agent compared to the base material.

The results of the thermogravimetric analysis (TGA) are presented in Figure 4. A key observation is that the onset of thermal decomposition for the membrane shifted from 380 °C down to 296 °C following its porous modification with cyclodextrin. This shift signifies a measurable decrease in the material’s thermal stability. This is because the specific surface area of porous membranes is significantly higher than that of dense-film membranes. During TGA measurement, this larger surface area exposes more imidazolium functional groups and polymer chain ends directly to the heating environment. These exposed sites (especially the thermally less stable organic imidazolium cations compared to the PVDF backbone) are more susceptible to early-stage degradation, such as the cleavage of alkyl side chains or the decomposition of the ionic liquid moieties, which is consistent with the initial weight loss step. Examination of the Thermogravimetric (TG) and Derivative Thermogravimetric (DTG) curves provides further insight. A new degradation peak is evident at 338 °C in the membrane containing residual porogen. This peak is ascribed to the thermal decomposition of the β-cyclodextrin. Importantly, this peak disappears after water treatment, confirming the successful removal of the template and the formation of a porous structure. Finally, the high-temperature degradation event observed at 449 °C is likely due to the breakdown of the primary PVDF polymer backbone.

### 3.3. Mechanical Characteristics

**Figure 5:**
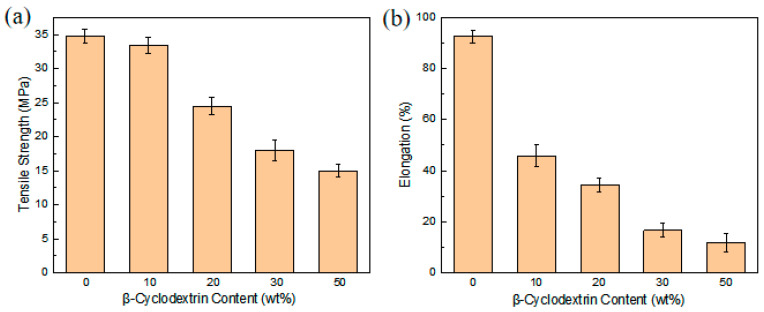
Tensile strength (**a**) and elongation (**b**) at break of PVDF-IL prepared with different pore-forming agents.

The mechanical performance of both dense and asymmetric porous membranes represents a vital consideration for their practical application. Test data summarized in Figure 5 demonstrate that a higher porogen concentration, which results in elevated porosity, corresponds with a reduction in tensile strength. The measured strength drops from 34.72 MPa for the non-porous membrane to 14.96 MPa for the sample with 50% porogen. Despite this decline, the value remains superior to the 13.87 MPa recorded for a Nafion 115 membrane. Fracture elongation is another essential metric for assessing a material’s ductility. The dense membrane exhibits a substantially greater elongation at break (92.52%) compared to its porous counterparts. This difference occurs because the perforation process compromises the structural continuity of the material. The introduction of a greater number of pores creates more sites that are vulnerable to stress, thereby diminishing the membrane’s overall ductility and mechanical strength.

### 3.4. Porosity, Water Uptake and Swelling Ratio

A comprehensive analysis of the physical characteristics of the asymmetric porous membranes was conducted, evaluating key parameters including thickness, void fraction, water uptake capacity, and volumetric swelling. These results are compiled in Table 1. The membranes were fabricated with controlled casting solution concentrations, revealing a direct correlation between rising cyclodextrin levels and increased membrane thickness. This effect is primarily attributed to the ability of cyclodextrin to increase solution viscosity, resulting in thicker casting layers.

Porosity stands as a fundamental property of porous membranes, offering direct insight into the volumetric extent of void formation and the overall porous architecture. Experimental data confirm a progressive enhancement in void fraction with greater porogen incorporation, verifying that elevated porogen concentrations effectively promote the creation of larger pore volumes. This increased void fraction subsequently strengthens ion exchange capacity, thus contributing to improved membrane conductivity.

The hydrophilicity of the membranes, quantified through water uptake measurements, offers valuable insight into interfacial contact with electrolytes. Analysis shows that the calculated porosity and water uptake both increase with β-cyclodextrin content. It is noted that the porosity value is an apparent one, referenced to the dry state. Membrane swelling upon hydration means the actual hydrated pore fraction is lower. However, the consistent upward trend across samples validates that the porogen successfully creates additional interconnected volume for water and ion transport, which is the key factor enhancing ionic conductivity. With a 30% porogen formulation, water uptake surpassed that of Nafion 115, attaining 26.89%, which indicates superior electrolyte-membrane interaction.

While improved hydration promotes better electrolyte contact and consequently enhances battery performance, excessive water uptake induces dimensional expansion through swelling, potentially degrading mechanical integrity and long-term viability. Accordingly, volumetric swelling ratios were measured. The dense membrane demonstrated negligible swelling, consistent with its poor hydration capacity. In contrast, swelling ratios increased progressively with porogen content, confirming the relationship between water absorption and dimensional change, as trapped water exerts greater expansive force within the porous matrix. Importantly, even at 50% porogen content, the membrane maintained a manageable swelling ratio of 17.80%, confirming its suitability for prolonged service in flow battery applications.

### 3.5. Ion Exchange Capacity and Conductivity Testing of Asymmetric Porous PVDF-IL Membranes

The IEC for the PVDF-IL and PVDF-IL-CD AEMs is presented in Figure S3. The observed increase in ion exchange capacity with higher β-cyclodextrin porogen content can be attributed to the enhanced accessibility of the imidazolium functional groups. In the dense PVDF-IL membrane, a fraction of the cationic groups are likely embedded within the hydrophobic polymer matrix, limiting their contact with the aqueous electrolyte during measurement. The introduction of a porous network via porogen leaching creates interconnected pathways that facilitate complete electrolyte permeation. This not only increases the effective surface area for ion exchange but also allows the electrolyte to reach previously inaccessible charged sites in the membrane bulk. Consequently, a greater proportion of the grafted imidazolium groups participate in the ion exchange process, resulting in a higher measured IEC.

**Figure 6:**
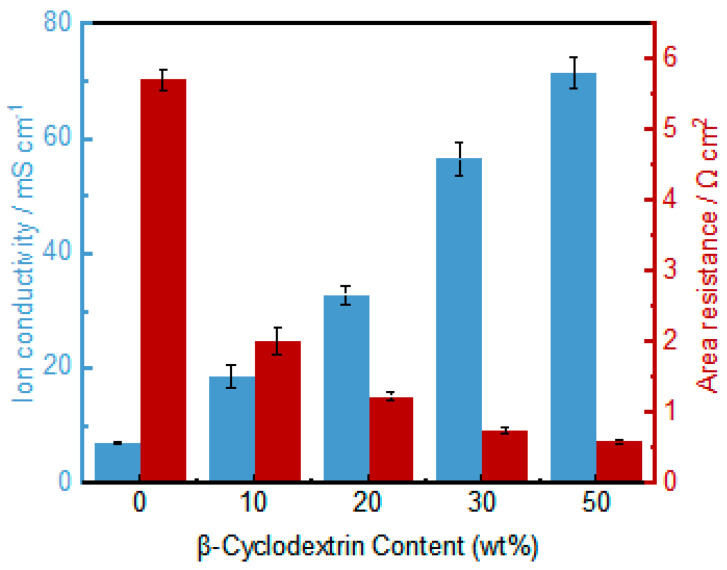
The ion conductivity and area resistance of the PVDF-IL and PVDF-IL-CD membranes.

Figure 6 presents the ionic conductivity measurements for membranes immersed in 3 M H_2_SO_4_ for 24 h. As shown in Figure 6, the area resistance of the AEM decreases significantly with increasing porogen concentration. The unmodified dense membrane, fabricated without any porogen, displayed a conductivity of merely 6.73 mS/cm. This constrained performance originates from the imidazolium functional groups within the dense structure, which primarily permit the exchange of anions like HSO_4_^−^ and SO_4_^2−^. The substantial Stokes radii of these anions hinder efficient ion transport, leading to suppressed conductivity. Enhanced porosity allows the membrane to interact with and hold a larger volume of electrolyte, thereby generating a greater number of HSO_4_^−^/imidazolium acid-base pairs. This increase directly contributes to more efficient ion transport mechanisms. The advancement of acid-base pair interaction through elevated void fraction further augments the membrane’s conductive properties. A continued upward trend in conductivity is observed with further increases in porosity, providing additional evidence that the introduction of a porous architecture effectively enhances the membrane’s ion conduction capability.

### 3.6. Permeability of Vanadium Ions

**Figure 7:**
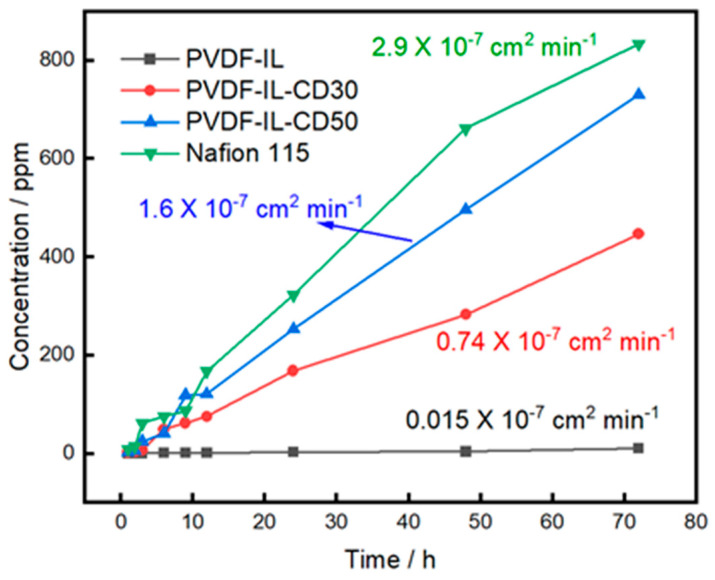
Vanadium permeability curves of PVDF-IL prepared with different pore-forming agents and Nafion membranes.

In vanadium flow battery systems, the evaluation of ion exchange membranes extends beyond ionic conductivity to include their capacity for vanadium ion rejection. Effective suppression of vanadium crossover markedly reduces self-discharge phenomena, thereby supporting sustained energy storage capacity. As illustrated in Figure 7, a time-dependent increase in vanadium ion concentration on the magnesium sulfate side confirms natural diffusion across the membrane. The dense membrane containing no porogen demonstrated exceptionally low ion permeability, with a calculated *p*-value of 0.015 × 10^−7^ cm^2^ min^−1^, representing merely 1/193 of the value measured for Nafion 115. However, introducing porosity substantially elevates vanadium ion transport rates. This effect arises from both the reduced effective membrane thickness and the creation of larger pores that offer less resistance to ionic migration. Nevertheless, the sample containing 50% porogen maintained a *p*-value of 1.6 × 10^−7^ cm^2^ min^−1^. The comparison of the thickness-normalized permeability coefficients (P) confirms that the PVDF-IL-based membranes possess a superior intrinsic resistance to vanadium ion transport compared to Nafion 115, despite their smaller thickness.

### 3.7. Antioxidant Properties

The durability of vanadium redox flow batteries is significantly limited by the highly oxidizing pentavalent vanadium ions, which induce severe chemical degradation in the composite membranes. This oxidative attack compromises the material’s structural integrity and diminishes the overall battery performance. Consequently, the development of composite membranes with superior oxidation resistance is essential for achieving long-term operational stability in these systems. In this study, the chemical stability of Nafion 115, PVDF-IL, and PVDF-IL-CD membranes was assessed via gravimetric analysis, recording the mass change for each sample following exposure to Fenton’s reagent (Table 2). The data reveal that, with increasing pore-forming agent content, oxidative stability progressively decreases. This decline is linked to the porous architecture, which substantially enlarges the surface area exposed to the strongly oxidizing solution, accelerating the degradation of both imidazolium moieties and the polymer backbone. Furthermore, interconnected pore channels facilitate the unimpeded diffusion of oxidizing species into the membrane’s interior, propagating degradation beyond the surface into deeper regions. Despite some mass loss, it is significant that the PVDF-IL-CD50 membrane retained more than 96% of its initial mass. This outcome is predominantly due to the inherent robustness of the PVDF matrix and the stabilizing effect of the imidazolium functionalities.

### 3.8. Single-Cell Testing of Asymmetric Porous PVDF-IL Separators

**Figure 8:**
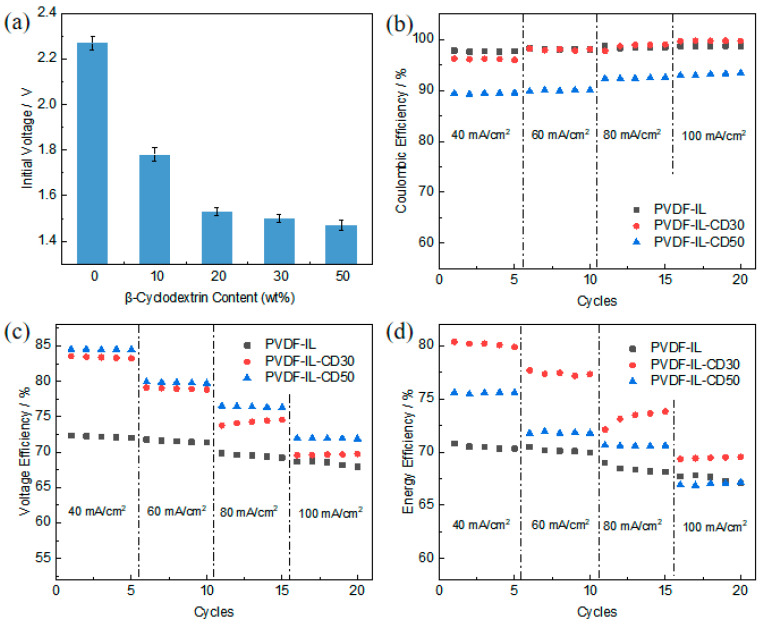
(**a**) Initial voltage of PVDF-IL prepared with different pore-forming agents; (**b**–**d**) charge–discharge test of PVDF-IL-CD40 and PVDF-IL-CD50 at different current densities.

The developed asymmetric porous separator showed highly favorable properties under static testing conditions. To evaluate its performance in practical application, the membrane was incorporated into an operational vanadium flow cell for galvanostatic cycling evaluation. Due to the substantial pore dimensions generated by the cyclodextrin porogen, the gains in specific surface area and resultant conductivity were constrained. Consequently, internal cell resistance caused certain separator samples to rapidly attain the predefined voltage limits during operation. A comparative analysis of the initial charging voltage subsequent to a full discharge (SOC = 0%) was performed to more precisely quantify conductivity enhancements. According to Figure 8a, separators fabricated with cyclodextrin exhibited a marked advancement in conductivity. The initial charge voltage measured after the cell was fully depleted dropped from 2.267 V to 1.453 V, signifying a substantial reduction in the separator’s resistance and verifying successful conductivity improvement.

The open-circuit voltage (OCV) profiles of VRFBs assembled with PVDF-IL and PVDF-IL-CD AEMs are presented in Figure S4. Initial discharge measurements reveal that the battery incorporating a Nafion 115 membrane achieves a higher starting voltage (1.53 V) compared to those using PVDF-based proton-conducting membranes, which can be attributed to Nafion’s inherently greater ion conductivity. However, after approximately 15 h of continuous operation, the VRFB with Nafion 115 exhibits a sharp decline in OCV. In contrast, cells constructed with the PVDF-IL and PVDF-IL-CD membranes demonstrate markedly extended stability, sustaining an OCV above 1.2 V for nearly 23 h—significantly longer than the Nafion-based system. This prolonged voltage maintenance indicates that VRFBs employing Nafion 115 experience more substantial self-discharge relative to those using the alternative AEMs. These OCV trends correspond well with prior permeability measurements, which show that vanadium-ion crossover through the PVDF-based AEMs is considerably lower than through Nafion 115. Therefore, within VRFB applications, the PVDF-IL and PVDF-IL-CD anion-exchange membranes exhibit a stronger capability to mitigate self-discharge.

To evaluate membrane performance in practical applications, the PVDF-IL-CD30 and PVDF-IL-CD50 separators were incorporated into a vanadium flow battery cell with an active area of 4 cm^2^ for single-cell testing. The resulting efficiency data are presented in Figure 8b–d. As illustrated in Figure 8b, the PVDF-IL-CD50 membrane consistently exhibits lower CE than the PVDF-IL-CD30 membrane across all current densities, aligning with its higher vanadium ion permeability due to greater porosity. This confirms the expected trade-off where increased porosity enhances ion crossover, thereby reducing CE. Figure 8c shows the VE outcomes. The PVDF-IL-CD50 membrane achieved higher VE than the PVDF-IL-CD30 counterpart, demonstrating that the developed porous architecture effectively reduces ionic resistance and improves voltage efficiency. As anticipated, VE for both membranes decreased with increasing current density due to amplified ohmic losses and overpotentials. EE, derived from the product of CE and VE, serves as the key indicator of overall membrane effectiveness. The trends shown in Figure 8d reveal that the PVDF-IL-CD30 membrane delivers the highest EE among the tested samples. This optimal performance stems from its better balance between maintaining acceptably high CE (through moderate vanadium blocking) and achieving significantly improved VE (through enhanced conductivity) compared to the dense or highly porous membranes.

Figure S5 presents the cycling performance, at 100 mA/cm^2^ over 200 cycles, of a cell employing a PVDF-IL-CD30 separator compared to one with a Nafion 115 membrane. As illustrated in Figure S5a, the PVDF-IL-CD30 cell maintained stable Coulombic, voltage, and energy efficiencies throughout the test, with no observable decline. This sustained performance indicates minimal degradation of the membrane’s ion-exchange functionalities within the strongly oxidizing V(V) electrolyte, a finding consistent with prior chemical stability assessments and confirming the material’s robust chemical resistance. Regarding capacity retention, Figure S5b reveals a marked difference between the two separators. After 200 cycles, the capacity of the cell with the PVDF-IL-CD30 membrane decayed to 70% of its initial value, whereas the cell with Nafion 115 experienced a more severe fade, retaining only 45% capacity. These results substantiate the superior vanadium ion blocking ability and enhanced long-term capacity retention of the PVDF-IL-CD30 membrane developed in this study.

## 4. Conclusions

Based on the research presented in this work, an asymmetric porous PVDF-IL membrane was successfully developed for vanadium flow batteries via a template-leaching method that employs the benign, water-soluble β-cyclodextrin as a porogen. The introduction of porosity significantly improved the membrane’s hydrophilicity, water uptake, and ion conductivity, addressing the low voltage efficiency of the original dense membrane. While increased porosity led to a reduction in mechanical strength and vanadium resistance, the modified membranes still exhibited superior vanadium blocking compared to commercial Nafion 115, especially at 30% porogen content. Across varied current densities, systematic testing established clear trends: CE declined with higher porogen content, whereas VE increased. The membrane with intermediate porosity (PVDF-IL-CD30) thus achieved the highest EE, demonstrating that tailored porosity effectively balances the inherent conductivity-selectivity trade-off in anion exchange membranes. Overall, this study demonstrates a feasible and environmentally benign approach to designing high-performance, asymmetric porous membranes. By combining the inherent Donnan exclusion of imidazolium-functionalized PVDF with a structurally engineered porous network, we provide a promising material strategy for advancing cost-effective and efficient membranes for durable flow battery applications.

## Figures and Tables

**Table 1:** Porosity, water uptake and swelling ratio.

	Thickness/μm	Porosity/%	Water Uptake/%	Swelling Ratio/%
Nafion 115	130	-	25.96 ± 1.48	10.06 ± 1.44
PVDF-IL	58	13.41 ± 1.13	7.76 ± 0.71	3.33 ± 0.55
PVDF-IL-CD10	55	27.61 ± 1.54	15.95 ± 0.85	5.45 ± 0.34
PVDF-IL-CD20	59	28.51 ± 1.32	17.68 ± 1.04	7.68 ± 0.81
PVDF-IL-CD30	62	32.44 ± 1.44	26.89 ± 1.42	14.70 ± 1.26
PVDF-IL-CD50	62	40.24 ± 1.63	31.79 ± 1.11	17.80 ± 1.31

Data showed in red are presented as mean ± standard deviation (n = 3).

**Table 2:** Oxidation resistance of PVDF-IL, PVDF-IL-CD and Nafion 115 membranes after exposure to Fenton reagent.

Membrane	S_fm_ (%)
PVDF-IL	0.96 ± 0.04
PVDF-IL-CD10	1.35 ± 0.08
PVDF-IL-CD20	1.72 ± 0.11
PVDF-IL-CD30	2.88 ± 0.09
PVDF-IL-CD50	3.36 ± 0.15
Nafion 115	0.78 ± 0.05

Data showed in red are presented as mean ± standard deviation (n = 3).

## Data Availability

The original contributions presented in this study are included in the article/Supplementary Materials. Further inquiries can be directed to the corresponding authors.

## Supplementary Materials

The following supporting information can be downloaded at https://www.mdpi.com/article/10.3390/ma19030583/s1, Figure S1: Cross-sectional SEM images of PVDF-IL-CD30 and PVDF-IL-CD50; Figure S2: N_2_ adsorption and desorption isotherms; Figure S3: The IEC of PVDF-IL and PVDF-IL-CD; Figure S4: OCV of the VRFB equipped with the PVDF-IL and PVDF-IL-CD membranes and Nafion115; Figure S5: (a) The cycling performance of the VRFBs with PVDF-IL-CD30 during 200 charge–discharge cycles at a current density of 100 mA cm^−2^. (b) Discharge capacity retention during 200 charge−discharge cycles at 100 mA cm^−2^.
