# Supplementary material for: Radiation-Induced Synthesis of Asymmetric Porous PVDF-g-PIL Membranes via β-Cyclodextrin Leaching for Vanadium Redox Flow Battery
Source: Materials (Basel). 2026 Feb 3;19(3):583. doi: 10.3390/ma19030583 (PMC12898593; doi:10.3390/ma19030583)
Supplement: Supplementary file 1 [file materials-19-00583-s001.zip › materials-4095104-supplementary.pdf]

Supporting Information

Radiation-induced synthesis of asymmetric porous PVDF-g-PIL membranes via β-cyclodextrin leaching for vanadium redox flow battery

Jiangtao Yu [a,b], Wenkang Li [c], Wei Niu [b], Manman Zhang [c], Junqing Bai [b], Pengtao Li [b], Liang Wang [b], Yuqing Cui [b], Shuanfang Cui [b], Xueyan Que [b *], Jun Ma [a *], Long Zhao [c *]

[a] School of Nuclear Science and Technology, University of Science and Technology of China, Hefei, Anhui 230026, China.

[b] Yangling Hesheng Irradiation Technologies Co., Ltd, Yangling, 712000, China.

[c] State Key laboratory of Advanced Electromagnetic Technology, School of Electrical and Electronic Engineering, Huazhong University of Science and Technology, Wuhan, 430074, China.

* Corresponding author :

Xueyan Que, E-mail address:xy_que@pku.edu.cn

Jun Ma, E-mail address:majun0502@ustc.edu.cn

Long Zhao, E-mail address: zhaolong@hust.edu.cn

Section 1. Method of tensile test

Initially, all membrane samples, including the Nafion 115 reference, were thoroughly dried in an oven at 80 °C for 24 h until a constant mass was achieved, defined by successive mass measurements differing by less than 0.2 mg. The thickness of each membrane was measured at three independent locations to ensure accuracy. Membranes were then precision-cut into a dumbbell geometry, carefully avoiding any edge imperfections. To prevent slippage during mechanical testing, double-sided adhesive was applied to both ends of each sample. Tensile tests were conducted at a constant crosshead speed of 50 mm min^{-1}. For each membrane formulation, three separate specimens were tested to ensure reproducibility, with data considered valid only if fracture occurred within the central gauge length.

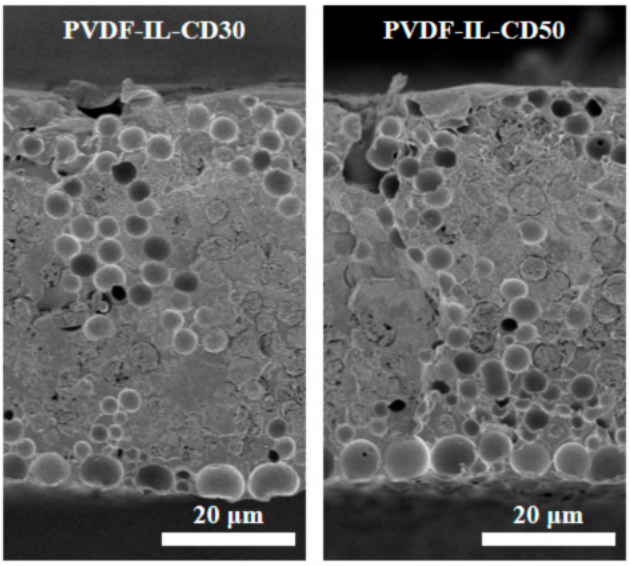

Figure S1 Cross-sectional SEM images of PVDF-IL-CD30 and PVDF-IL-CD50.

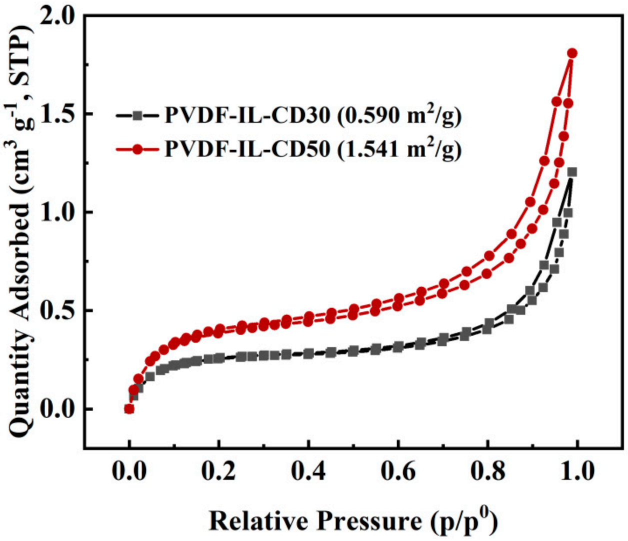

Figure S2. N_2 adsorption and desorption isotherms.

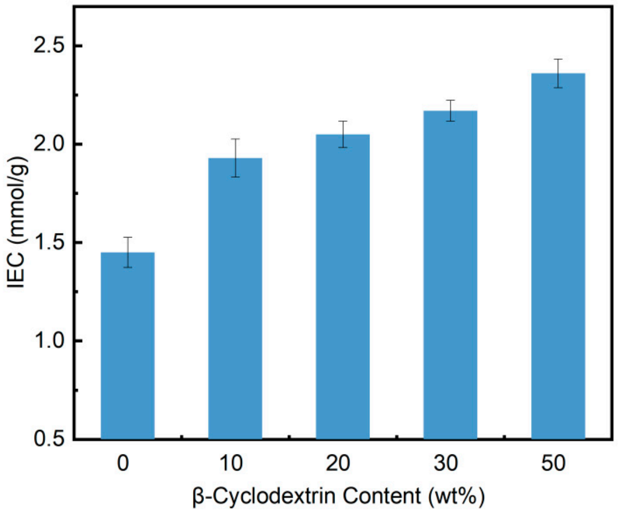

Figure S3 The IEC of PVDF-IL and PVDF-IL-CD

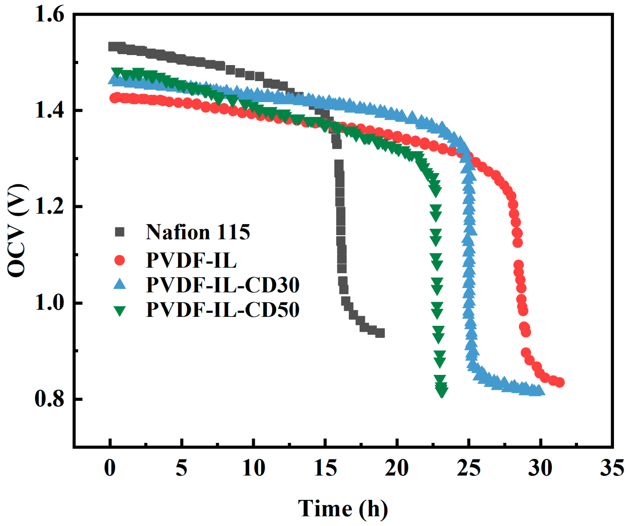

Figure S4 OCV of the VRFB equipped with the PVDF-IL and PVDF-IL-CD membranes and Nafion115.

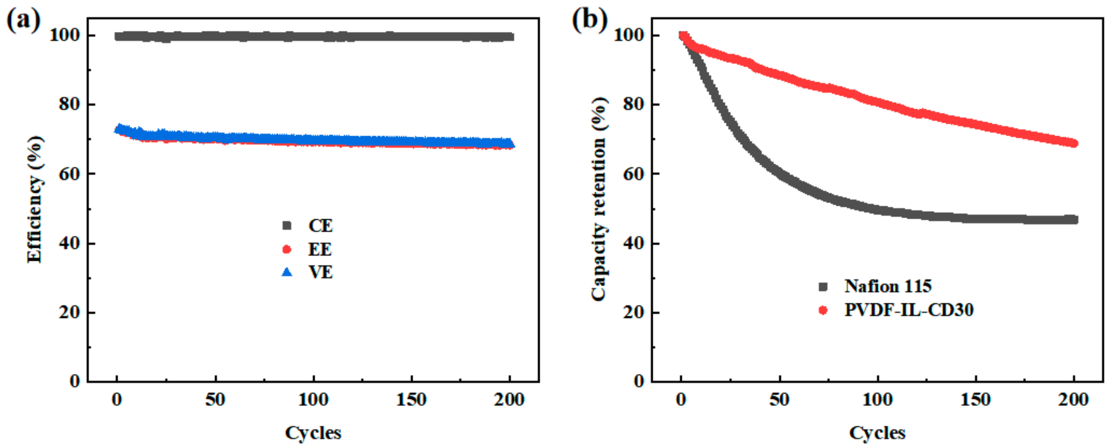

Figure S5 (a) The cycling performance of the VRFBs with PVDF-IL-CD30 during 200 charge–discharge cycles at a current density of 100 mA cm^{-2}. (b) Discharge capacity retention during 200 charge−discharge cycles at 100 mA cm^{-2}.
